# End‐diastolic force pre‐activates cardiomyocytes and determines contractile force: role of titin and calcium

**DOI:** 10.1113/JP277985

**Published:** 2019-07-30

**Authors:** Aref Najafi, Martijn van de Locht, Maike Schuldt, Patrick Schönleitner, Menne van Willigenburg, Ilse Bollen, Max Goebel, Coen A. C. Ottenheijm, Jolanda van der Velden, Michiel Helmes, Diederik W. D. Kuster

**Affiliations:** ^1^ Amsterdam UMC, Vrije Universiteit Amsterdam, Physiology Amsterdam Cardiovascular Sciences de Boelelaan 1117 1081 HZ Amsterdam the Netherlands; ^2^ Netherlands Heart Institute PO box 19258 3501 DG Utrecht the Netherlands; ^3^ Ionoptix de Boelelaan 1108 1081 HV Amsterdam the Netherlands; ^4^ CytoCypher de Boelelaan 1108 1081 HV Amsterdam the Netherlands

**Keywords:** contractility, titin, pre‐activation, Frank‐Starling mechanism, calcium, RBM20, cardiomyocyte

## Abstract

Titin functions as a molecular spring, and cardiomyocytes are able, through splicing, to control the length of titin. We hypothesized that together with diastolic [Ca^2+^], titin‐based stretch pre‐activates cardiomyocytes during diastole and is a major determinant of force production in the subsequent contraction. Through this mechanism titin would play an important role in active force development and length‐dependent activation. Mutations in the splicing factor RNA binding motif protein 20 (RBM20) result in expression of large, highly compliant titin isoforms. We measured single cardiomyocyte work loops that mimic the cardiac cycle in wild‐type (WT) and heterozygous (HET) RBM20‐deficient rats. In addition, we studied the role of diastolic [Ca^2+^] in membrane‐permeabilized WT and HET cardiomyocytes. Intact cardiomyocytes isolated from HET left ventricles were unable to produce normal levels of work (55% of WT) at low pacing frequencies, but this difference disappeared at high pacing frequencies. Length‐dependent activation (force–sarcomere length relationship) was blunted in HET cardiomyocytes, but the force–end‐diastolic force relationship was not different between HET and WT cardiomyocytes. To delineate the effects of diastolic [Ca^2+^] and titin pre‐activation on force generation, measurements were performed in detergent‐permeabilized cardiomyocytes. Cardiac twitches were simulated by transiently exposing permeabilized cardiomyocytes to 2 µm Ca^2+^. Increasing diastolic [Ca^2+^] from 1 to 80 nm increased force development twofold in WT. Higher diastolic [Ca^2+^] was needed in HET. These findings are consistent with our hypothesis that pre‐activation increases active force development. Highly compliant titin allows cells to function at higher diastolic [Ca^2+^].

## Introduction

During exercise and increased stress, cardiac work is adjusted to meet the increased demands of the body. Work of the heart is modulated by changing contractile force as well as relaxation capacity. Cardiac contraction and relaxation depend on preload (the Frank–Starling mechanism, i.e. length‐dependent activation), inotropic state (sympathetic drive) and afterload (peripheral resistance). The giant protein titin is a central player in cardiac muscle relaxation and contributes to impaired relaxation in cardiac disease (LeWinter & Granzier, 2013; Linke & Hamdani, 2014).

Titin's role in regulating passive tension is well‐established. In the heart, titin is spliced into two isoforms, the stiff N2B isoform and the longer and more compliant N2BA isoform. Changes in titin isoform composition and phosphorylation occur during development of cardiac disease, which contribute to altered cardiac performance (LeWinter & Granzier, 2013; Linke & Hamdani, 2014). Titin is not only a passive spring element involved in regulating muscle stiffness, but can also influence cardiac contraction. Studies that elucidated the role of titin in cardiac contractility have mainly been performed in murine models. In the Greaser laboratory a rat model was identified with very large titin isoforms (Greaser *et al*. 2008). Later it was established that a mutation in the splicing factor RBM20 caused these ‘giant’ titin proteins (Guo *et al*. 2012). In addition, mutations in RBM20 have been identified as a cause for dilated cardiomyopathy in patients, and were associated with expression of highly compliant ‘giant’ titin isoforms (Brauch *et al*. 2009; Guo *et al*. 2012; Beqqali *et al*. 2016). While large compliant titin isoforms reduce passive tension (Greaser *et al*. 2008; Patel *et al*. 2012; Methawasin *et al*. 2014) and thus may improve cardiac relaxation (Methawasin *et al*. 2014), expression of giant titin isoforms has been associated with reductions in maximal force (Patel *et al*. 2012; Mateja *et al*. 2013; Methawasin *et al*. 2014) and length‐dependent activation (Patel *et al*. 2012; Methawasin *et al*. 2014). Moreover, reduced RBM20 expression, either via a mutation or knock‐out, leads to ventricular dilatation (Brauch *et al*. 2009; Guo *et al*. 2012) and decreased survival in rodents (Brauch *et al*. 2009). Interestingly, the effects seem to be dose‐dependent, as heterozygotes have intermediate effects on titin length, passive force, slack sarcomere length and length‐dependent activation (Methawasin *et al*. 2014). Having a large compliant titin isoform might also confer some benefits, as exercise capacity was increased in heterozygote RBM20 mutant mice (Methawasin *et al*. 2014).

To reconcile the low force production under basal conditions with the increased exercise capacity in rodents with compliant titin, we hypothesized that it is the end‐diastolic force that determines the amount of force generation. To test this, we measured the maximum work generating capacity in intact cardiomyocytes isolated from RBM20^+/−^ (HET) and wild‐type (WT) rats at different pacing frequencies and different pre‐loads. By using membrane‐permeabilized cardiomyocytes, we defined the effect of increasing diastolic calcium levels on myofilament force production as a second source of pre‐activation, to find out how titin and diastolic calcium levels complement each other.

## Methods

### Cell isolation

All animal experiments were performed under approval of the Animal Care and Use Committee of the VU University Medical Centre (Amsterdam) and conform to the guidelines from Directive 2010/63/EU of the European Parliament on the protection of animals used for scientific purposes (animal approval number Fys 11‐02). The rats were housed in a controlled environment with a 12 h light–dark cycle at room temperature (22°C) and had unlimited access to food. All animals are from a hybrid strain consisting of 50% Brown Norway–25% Fisher 344–25% Sprague–Dawley (Greaser *et al*. 2008).

Adult rat cardiomyocytes were isolated from WT (*N* = 7) and RBM20 HET (*N* = 7) male and female rats, weighing ∼300 g as described before (Helmes *et al*. 2016; van Deel *et al*. 2017). Anaesthesia was induced in rats by 8% sevoflurane and maintained with 1–2.5% sevoflurane. The isolated cardiomyocyte measurements were performed in a Tyrode solution (composition (mmol l^−1^): 130 NaCl, 5.4 KCl, 3 sodium pyruvate, 25 Hepes, 0.5 MgCl_2_, 0.33 NaH_2_PO_4_, 22 glucose) containing 1.8 mmol l^−1^ Ca^2+^ at 37°C.

### Titin isoform composition analysis

Titin isoform composition was analysed in rat left ventricular homogenates using a vertical Hoefer SE600 gel system (Hoefer Inc., Richmond, CA, USA) with a 1% agarose gel (1% w/v Sea Kem Gold agarose (Lonza, Rockwell, ME, USA), 30% v/v glycerol, 50 mmol l^−1^ Tris‐base, 0.384 m glycine, and 0.1% w/v SDS), as previously described (Warren *et al*. 2003; Bollen *et al*. 2017b). Samples were normalized against myosin heavy chain content and loaded in triplicates.

### Protein analysis

Cardiac troponin I (cTnI) phosphorylation and phospholamban (PLN) expression level and phosphorylation levels were determined in isolated cardiomyocytes (*N* = 6 WT, *N* = 5 HET) as described before (Najafi *et al*. 2016). In short, unphosphorylated cTnI was separated from mono‐ and bis‐phosphorylated forms by Phos‐tag SDS‐PAGE followed by western blot. cTnI was visualized upon incubation with cTnI antibody (MA1‐22700, Thermo Fisher Scientific, Waltham, MA, USA). PLN expression and phosphorylation were studied using site‐specific antibodies (total PLN, antibody A010‐14; P‐Ser16 PLN: antibody A010‐12AP, Badrilla Ltd., Leeds, UK). Total PLN levels were normalized to α‐actinin and PLN‐Ser16 phosphorylation was normalized to total PLN levels.

### Intact cardiomyocyte work loop measurements

Single intact cardiomyocytes were attached to the cantilever of the force transducer and to the piezo translator as described previously (Helmes *et al*. 2016). For this paper a new version of the interferometry‐based force transducer OptiForce was developed (the OptiForce V3, IonOptix, Westwood, MA, USA). While still using interferometry to measure the cantilever deflection, we applied a modulating technique (McGarrity & Jackson, 1994) that allowed us to linearize the force signal in real time. This allowed us to move to more compliant probes (from 20 N m^−1^ to 2 N m^−1^) while increasing the dynamic range. This improved the signal to noise ratio of the system approximately fivefold compared to the previous version. The 2 N m^−1^ stiffness probes allowed a force resolution of better than 1 nN, with a linear range of approximately 5 µN.

The tips (10 µm glass fibre) of the force transducer and piezo were coated with an aluminum silicate suspension (IonOptix pre‐coat). The pre‐coat was air‐dried after which they were dipped in MyoTak (IonOptix). Finally, the cardiomyocyte was attached to the pre‐coated and MyoTak‐coated glass fibre tips of the force transducer and piezo.

To mimic the cardiac pressure–volume relationship at the cellular level with an analogous force–length relationship, we implemented a feedback control system that, by modulating the cardiomyocyte length, can bound the force generated by the cardiomyocyte between a predefined preload and afterload. Preload and afterload are nomenclature in whole heart measurements and indicate the volume at end‐diastole and the aortic pressure against which the blood is ejected during systole, respectively. Here they refer to the target force levels of the feedback control during the diastolic and systolic phase of the myocyte contraction, respectively. The work loop protocol was performed as described previously (Helmes *et al*. 2016). Briefly, for each pre‐load (we tested four levels for each cell), the afterload was varied over a range so as to capture the pre‐ and afterload combination that produced maximum work. This was done at pacing frequencies of 1, 2, 4, 6 and 8 Hz.

### Pre‐activation protocol of membrane‐permeabilized cardiomyocytes

From a small piece of left ventricle, cardiomyocytes were isolated as described previously (van der Velden *et al*. 1998). We improved the cell gluing process by using shellac (wax‐free Sigma‐Aldrich, St Louis, MO, USA, 78471; 0.07 mg ml^−1^ 70% ethanol), instead of the usual silicone glue. This resulted in a much stronger attachment while the glue cures almost instantly, speeding up the experimental process. The single membrane‐permeabilized cardiomyocyte was glued to the same force measuring system as the intact myocytes. The high signal‐to‐noise ratio and the high dynamic range of this force transducer allowed us to measure both the small changes in diastolic force and the much bigger systolic forces (see Fig. 4).

The use of a rapid switch perfusion system (VC‐77CSP, Warner Instruments, Hamden, CT, USA) using pipettes pulled from theta tubing allowed rapid switching (<10 ms) between perfusion solutions. Here the use of an optical force probe led to complications. The calcium propionate we normally use to make our activating solution absorbs near‐infrared light, interfering with the force measurements. We therefore used calcium chloride for our solutions, which greatly reduced this effect. We also engineered the system to keep the solution flowing between the tip of the optical fibre and the cantilever constant. The flow chamber was attached to the chassis of the microscope, so it was stationary with respect to the force probe. The narrow flow channel allowed us to use a background flow matched to the flow from the theta tubing, so that the flow throughout the channel was laminar, preventing backflow of the solution with the high [Ca^2+^]. The temperature of the flow chamber could be controlled using a Peltier element. For these experiments it was kept at 20°C. The coverslip with the cells can move freely under the flow chamber, so in spite of the confined space of the flow chamber, the cells can be selected from a large area.

The experimental protocol that was performed had five sets of activations, each exposing the myocyte to a 2 µm free calcium solution for 1 s, to mimick the time‐limited nature of cardiac contractions. Each set had a different diastolic [Ca^2+^] (1, 80, 160, 250 and 400 nm Ca^2+^), while keeping the activating [Ca^2+^] constant (2 µm Ca^2+^) (see Fig. 4). After three cycles of activation and relaxation with 2 µm Ca^2+^ and 1 nm Ca^2+^, the myocyte was stretched (from 1.8 µm to 2.0 µm resting length for WT and from 2.0 µm to 2.2 µm after which the protocol was repeated. Subsequently the diastolic [Ca^2+^] was increased from 1 nm to 80 nm, whereas the activating [Ca^2+^] remained the same (i.e. 2 µm). We repeated this procedure also for diastolic calcium solutions with 160, 250 and 400 nm Ca^2+^, while the activation solution contained 2 µm Ca^2+^. The forces were normalized by averaging the force of the first three cardiomyocyte activations (i.e. 1 nm as diastolic and 2 µm Ca^2+^as activation solution) and set as 1 at time 1 s from the switch of the pipet from diastolic to the activating calcium. We investigated if increasing diastolic calcium might result in a higher active force at 2 µm Ca^2+^ relative to the first activation (i.e. 1 nm Ca^2+^).

### Data analysis

Both the work loop data and the force data from the permeabilized cells were analysed using a modified version of Transient Analysis Tools (CytoCypher BV, Amsterdam, the Netherlands).

Further data analysis and statistics were performed using Prism version 7.0 (GraphPad Software, Inc., La Jolla, CA, USA). Data are presented as means ± SEM of all single cardiomyocytes per rat group. Data were tested for normality by Kolmogorov–Smirnov normality test. When data were distributed normally and in the case of testing one variable in more than two groups, the groups were compared using one‐way analysis of variance (ANOVA); in the case of two or more variables, the data were compared using a two‐way ANOVA. If a significant value in two‐way ANOVA was detected, a Holm–Sidak multiple comparison *post hoc* test was performed to identify significance within multiple groups. Significance was accepted when *P* < 0.05. *N* indicates the number of animals; *n* indicates the number of cardiomyocytes measured.

## Results

### Longer titin, but no changes in cTnI and PLN phosphorylation in HET compared to WT

**Figure 1:**
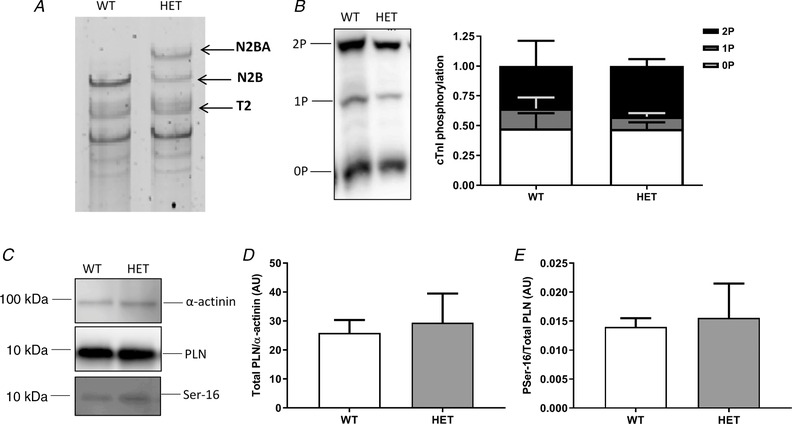
Protein analysis in isolated cardiomyocytes from WT and HET hearts *A*, in HET cardiomyocytes a longer titin isoform is seen. *B*, Phos‐tag analysis of cardiac troponin I was performed to assess its phosphorylation state. No difference in cTnI phosphorylation was seen between WT (*N* = 6) and HET (*N* = 5). *C*, immunoblot analysis was performed to measure PLN expression levels and phosphorylation. *D* and *E*, no difference was seen in expression levels (*D*) and phosphorylation at Ser16 (*E*) between WT (*N* = 6) and HET (*N* = 5). Data are shown as means ± SEM.

Titin isoform expression was determined in freshly isolated cardiomyocytes. As previously reported, longer titin isoforms were found in the HET compared to WT cardiomyocytes (Fig. 1 *A*). Because phosphorylation of cTnI and PLN is an important regulator of cardiac contractility, we studied their phosphorylation levels. Using Phos‐tag analysis to assess cTnI phosphorylation, we found no difference in cTnI phosphorylation between the two groups (Fig. 1 *B*). PLN expression and phosphorylation were measured by immunoblot analysis and no difference in PLN expression levels (Fig. 1 *C*) and phosphorylation (Fig. 1 *D*) was observed.

### Reduced work output in RBM20 cardiomyocytes only at low pacing frequencies

**Figure 2:**
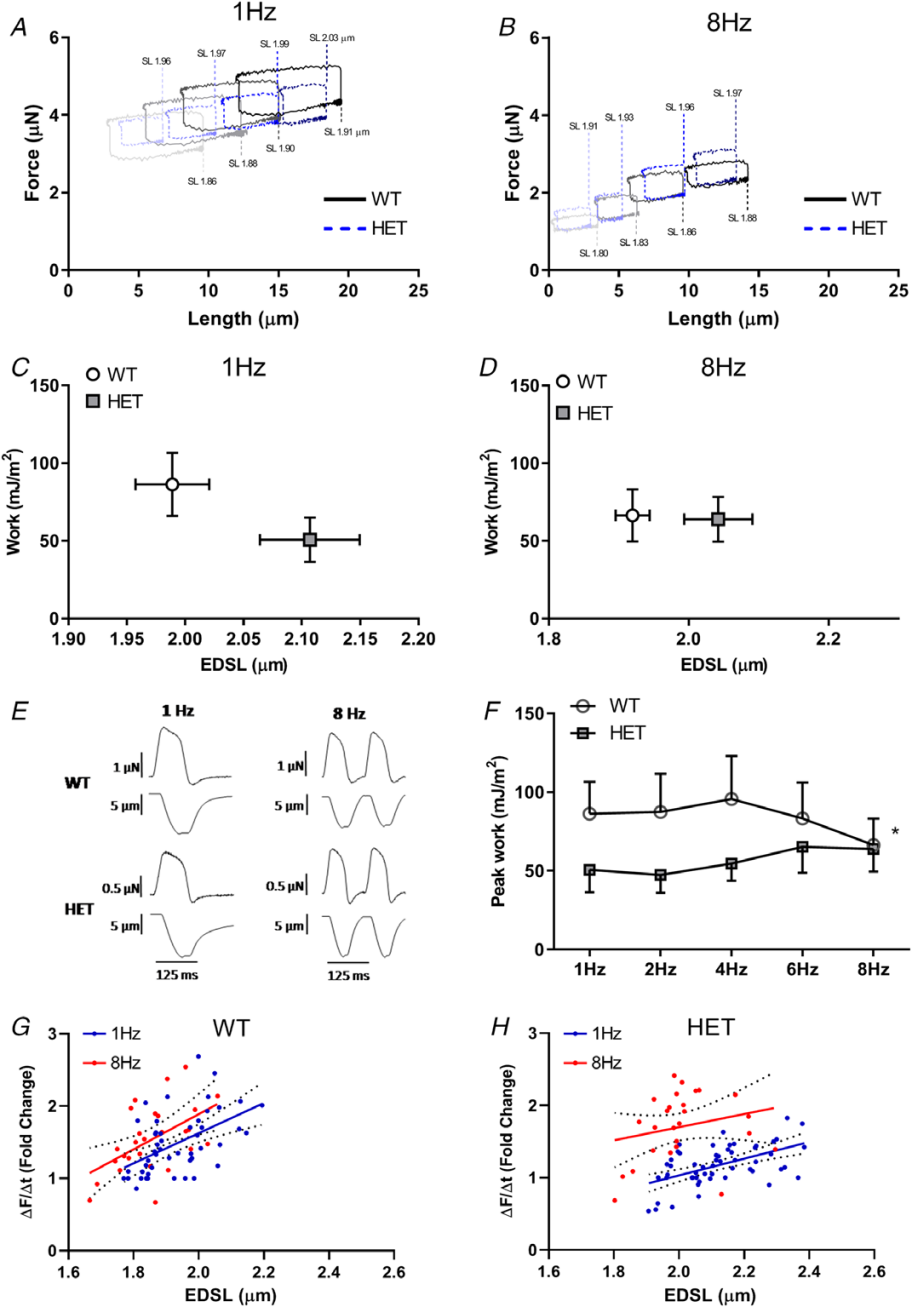
Maximal work in RBM^+/−^ cardiomyocytes only reduced at low pacing frequencies *A* and *B*, example tracings of work loops from WT and HET cardiomyocytes at 4 different preloads paced at 1 Hz (*A*) or 8 Hz (*B*). *C* and *D*, peak work at 1 Hz (*C*) and 8 Hz (*D*) was plotted against the end‐diastolic SL at which peak work was achieved (*n* = 8). End‐diastolic sarcomere length (EDSL) was always higher in HET. *E*, example tracings of force and length changes of contractions at 1 and 8 Hz for WT and HET. *F*, peak work over the range of pacing frequencies measured. Lower peak work in HET compared with WT only at low pacing frequencies. ^*^ *P* < 0.05. *G* and *H*, kinetics of contraction during the isometric contraction phase at different preloads were plotted for WT (*G*) and HET (*H*) cardiomyocytes (for each group *n* = 7). The force scales in *A* and *B* are relative; it was not possible to reliably measure and track zero force after the myocytes were attached.

To explain the previously reported higher exercise capacity (Methawasin *et al*. 2014), we performed work loop experiments in isolated adult intact rat cardiomyocytes at different pacing frequencies (1–8 Hz). By stretching the cells to four different preloads and constantly changing the afterload, we could explore the parameter space of pre‐load, afterload and work and could establish the pre‐ and afterload combination that produced maximal work (for details, see Helmes *et al*. 2016). This was performed at each pacing frequency for both WT and HET (Fig. 2 *A* and *B*). Cells that underwent this pacing protocol underwent no appreciable run‐down, as the fold change of the mean power produced at 1 Hz at the beginning of the protocol *versus* the end (after pacing rate was increased to 2, 4, 6 and 8 Hz before going back to 1 Hz) was 1.11 ± 0.2 for WT and 0.90 ± 0.15 for RBM20 (data displayed at means ± SEM for 7 WT and 6 RBM20 cells). At low pacing frequencies the maximal work was significantly lower in HET compared to WT cells (Fig. 2 *C*). With the same pre‐load, WT cells operated at lower sarcomere length (SL) than HET. When increasing the pacing frequency to 8 Hz, the maximal work was no longer different between the groups, although the end diastolic SL was still longer in HET (Fig. 2 *D*) for each given pre‐load. When looking at the range of frequencies from 1 to 8 Hz, the maximum work output per contraction decreased in WT cardiomyocytes, while in HET, maximum work per contraction increased with each increase in pacing frequency (Fig. 2 *F*, *P* < 0.05). Kinetics of contraction during the isometric phase were plotted for each preload in WT and HET cardiomyocytes paced at 1 and 8 Hz. As expected WT cells had faster contraction and showed a greater increase in kinetics upon changes in SL (Fig. 2 *G* and *H*). HET cardiomyocytes showed a greater increase in kinetics when pacing rate was increased from 1 to 8 Hz (Fig. 2 *H*).

### Systolic force development is dependent on end‐diastolic force

**Figure 3:**
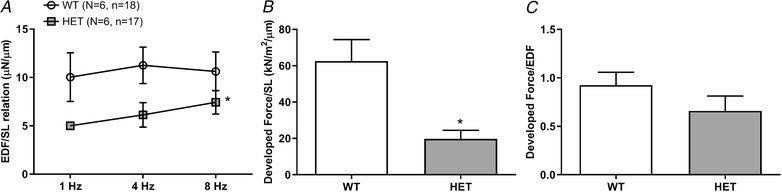
End‐diastolic force not SL determines length‐dependent activation During the work loop protocol cells are stretched to different preload (end‐diastolic force; EDF), while SL is recorded. *A*, the EDF to SL relationship shows that HET cardiomyocytes require much less force to be stretched, which holds true at different pacing frequencies (*N* = 6, *n* = 18 WT; *N* = 6, *n* = 17 HET)*. B*, as developed force is measured during the subsequent contraction, the developed force to end‐diastolic SL relationship can be measured. This relationship is a measure of length‐dependent activation. This is severely blunted in HET cardiomyocytes (*N* = 6, *n* = 17 WT; *N* = 6 *n* = 9 HET)*. C*, however, if EDF is plotted against subsequent developed force, only a small non‐significant decrease is seen in HET cardiomyocytes compared with WT (*N* = 6, *n* = 21 WT; *N* = 6, *n* = 10 HET).

For each contraction the end‐diastolic force (EDF), end‐diastolic sarcomere length (EDSL) and force development were measured. Because cardiomyocytes are stretched to different preloads during the work loop protocol, we could determine the EDF/EDSL ratio, and how much an increase in SL and in EDF influences developed force on the subsequent contraction. As expected, the EDF/SL relation was significantly lower in HET than in WT cardiomyocytes at the different pacing frequencies (Fig. 3 *A*). Previous articles reported blunted length‐dependent activation (LDA) in this model (Patel *et al*. 2012; Methawasin *et al*. 2014). Here we found that the developed force–SL relation was also severely blunted (Fig. 3 *B*). Thus with an equivalent increase in SL, the increase in force production is much smaller in HET compared to WT. However, the developed force–EDF relationship was not significantly different between WT and HET cardiomyocytes (Fig. 3 *C*). This means that a similar increase in EDF leads to a similar increase in force production in WT and HET. These data indicate that, regardless of titin isoform, the stretch‐mediated EDF is the major determinant of the subsequent systolic force development.

### Pre‐activation of permeabilized cardiomyocyte increases force development

**Figure 4:**
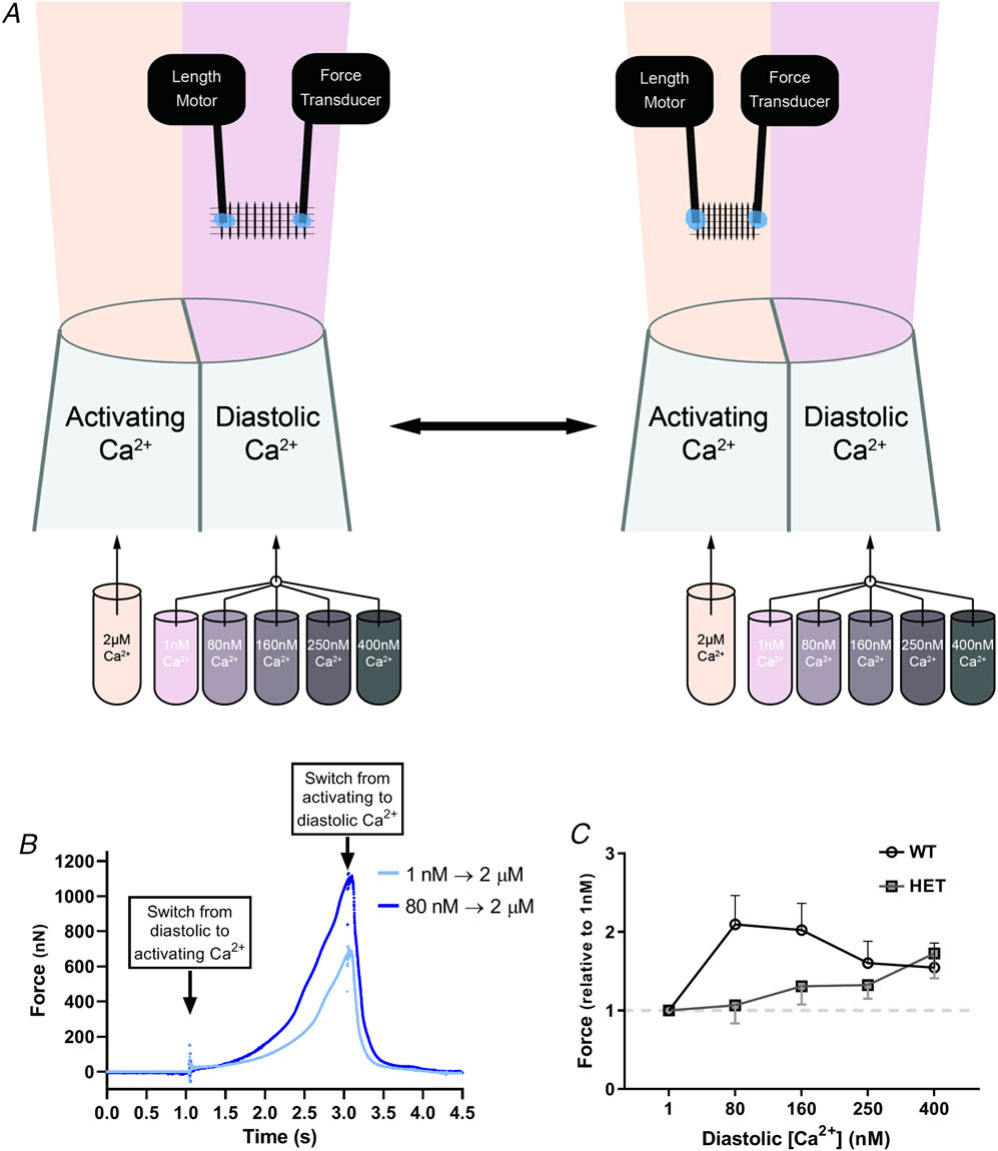
Pre‐activation of HET cardiomyocytes occurs at higher diastolic Ca^2+^ *A*, membrane‐permeabilized cardiomyocytes were glued between a force transducer and a length motor. Using a theta‐glass perfusion system, we could rapidly switch solutions to mimic a cardiac contraction. During the protocol we use one activation solution (2 µm Ca^2+^) and five different diastolic solutions (ranging from 1 to 400 nm Ca^2+^). Cells were perfused with diastolic solution until the force trace is stable and the pipette was rapidly switched so that the cell was exposed to activation solution for 1 s, before moving back to diastolic solution. After repeating this 3 times, the diastolic Ca^2+^ was changed to a different concentration. *B*, example tracing from a WT permeabilized cardiomyocyte undergoing the pre‐activation protocol. A permeabilized cardiomyocyte was constantly perfused with 1 nm diastolic calcium solutions and then exposed to 2 s perfusion of activating Ca^2+^ (2 µm). The same cell was then perfused with 80 nm diastolic Ca^2+^ until the force signal was stable. When switching to the same activating Ca^2+^, almost double the force was produced. For measurements of active force production at different diastolic Ca^2+^, background force that was produced by perfusion with each diastolic Ca^2+^ solution used was subtracted from force that was generated in the subsequent contraction. *C*, averaged data for active force development in WT (at 1.8 µm SL) and HET (at 2.0 µm SL) cardiomyocytes pre‐activated by different diastolic calcium concentrations. WT *N* = 6, *n* = 18; HET: *N* = 6, *n* = 11.

Based on the previous result it is to be expected that increasing EDF by increasing diastolic [Ca^2+^] will also increase force development. To be able to control diastolic and systolic [Ca^2+^] and test this hypothesis, we measured force development by detergent‐permeabilized, mechanically isolated cardiomyocytes. Transient force development as occurs in intact cardiomyocytes was mimicked using a fast solution‐switching set‐up (Fig. 4 *A*). With this set‐up we exposed myocytes to activation calcium (2 µm Ca^2+^) for 1 s. To pre‐activate the permeabilized cardiomyocyte, the diastolic [Ca^2+^] was increased stepwise. Increasing diastolic [Ca^2+^] from 1 nm to 80 nm increased the subsequent force production twofold in WT cardiomyocytes (Fig. 4 *B*). The maximal effect of pre‐activation was reached at 80 nm as a further increase in diastolic calcium reduced force development, mainly by increasing diastolic force. In HET cardiomyocytes, a different pre‐activation response was seen. Almost no pre‐activation takes place at low diastolic [Ca^2+^] (Fig. 4 *C*). In these cells, the maximal effect on pre‐activation was seen at 400 nm Ca^2+^.

## Discussion

The role titin plays in regulating passive stiffness has been extensively studied (Linke *et al*. 1994; Granzier & Irving, 1995). Cardiac muscle cells are able to decrease passive stiffness by favouring production of the longer more compliant N2BA isoform over the stiff N2B isoform (Cazorla *et al*. 2000; Freiburg *et al*. 2000). Levels of N2BA and N2B in the heart are regulated by splicing factors (Labeit & Kolmerer, 1995; Freiburg *et al*. 2000; Bang *et al*. 2001). Another mechanism to decrease passive stiffness is increasing protein kinase A/protein kinase G/extracellular signal‐regulated kinase 2 phosphorylation or decreasing protein kinase C phosphorylation (Hamdani *et al*. 2017). The *in vivo* consequences of decreasing titin stiffness have been extensively studied since the finding from Greaser *et al*. (2008) who identified a rat model that expressed a large highly compliant isoform. It was later identified that mutations in the splicing factor RBM20 caused these compliant titin molecules (Guo *et al*. 2012) and that mutations in the RBM20 gene can cause dilated cardiomyopathy (Brauch *et al*. 2009; Guo *et al*. 2012). These compliant titin molecules lead to a severe reduction in passive force in cardiomyocytes in rats (Greaser *et al*. 2008; Patel *et al*. 2012), mice (Methawasin *et al*. 2014) and humans (Beqqali *et al*. 2016). The effects on active force development, however, are less clear. Maximal force production in permeabilized cardiomyocytes was reduced in RBM20 knock‐out (KO) rats (Patel *et al*. 2012) and mice (Methawasin *et al*. 2014), but not in HET mice (Methawasin *et al*. 2014). Echocardiography‐derived fractional shortening was not changed in HET and KO rats (Guo *et al*. 2012), although both of them showed dilatation and reduced survival. In mice, only homozygous loss of RBM20 resulted in reduced fractional shortening, but both HET and KO mice showed reduced *in vivo* contractility (measured as end‐systolic elastance) (Methawasin *et al*. 2014). In myofibril experiments it was shown that kinetics of contraction (*k*_act_ and *k*_tr_) of skeletal muscle are reduced in KO rats (Mateja *et al*. 2013). Furthermore, reductions in length‐dependent activation were seen in rats (Patel *et al*. 2012), mice (Methawasin *et al*. 2014) and human (Beqqali *et al*. 2016). While mutations in RBM20 resulted in reduced contractility and dilated cardiomyopathy, RBM20 HET mice showed increased exercise capacity (Methawasin *et al*. 2014; Bull *et al*. 2016). Furthermore, in an experimental hypertensive model in mice, increasing titin compliance, through inducible functional knockout of RBM20, corrects subsequent diastolic dysfunction and increases exercise capacity (Methawasin *et al*. 2016).

Here we show that force development of cardiomyocytes is largely determined by the end‐diastolic force. The end‐diastolic force is a combination of passive and residual active force and the interplay between them determines the force development in systole. We show that rat cardiomyocytes with compliant titin produce less work at low pacing frequencies when end‐diastolic forces are low. This is comparable with the reduced contractility found in RBM20 HET mice (Methawasin *et al*. 2014). But the difference in work output is no longer present at higher pacing frequencies. Increasing pacing frequency leads to an increase in diastolic Ca^2+^ levels (Frampton *et al*. 1991; Layland & Kentish, 1999; Gattoni *et al*. 2016). Diastolic calcium increases from 94–105 nm at 1 Hz (Frampton *et al*. 1991; Gattoni *et al*. 2016) to 202–220 nm at 4 Hz (Dibb *et al*. 2007; Gattoni *et al*. 2016) to 255 nm at 8 Hz (Dibb *et al*. 2007). While WT cardiomyocytes show an increased active force production only when increasing diastolic [Ca^2+^] from 1 to 80 nm, active force production only decreases upon increasing diastolic [Ca^2+^]. As diastolic calcium concentrations at 1 Hz is already higher than 80 nm, WT cells do not profit from the increased diastolic Ca^2+^ at higher pacing frequencies. Here we have shown that HET permeabilized cardiomyocytes do produce more active force if they are exposed to high diastolic calcium levels seen at high pacing frequencies (Fig. 4 *C*). This might even be exacerbated by the increased diastolic [Ca^2+^] concentration that is seen in mouse RBM heterozygote KO cardiomyocytes (van den Hoogenhof *et al*. 2018), although we did not observe a higher basal diastolic [Ca^2+^] in WT *vs*. HET cells (data not shown). Our measurements indicate that high diastolic calcium can overcome the reduced contractility of HET cardiomyocytes. The latter observation can explain the contradictory findings of reduced basal contractility and increased exercise capacity in RBM20‐deficient models (Methawasin *et al*. 2014; Bull *et al*. 2016).

If compliant titin leads to less pre‐activation, stiffer titin should lead to increased contraction under basal conditions. The effect of increasing titin stiffness on cardiac function has been assessed by removing part of the titin gene, resulting in shorter and thus stiffer titin proteins. Mice with stiff titin isoforms showed increased end‐diastolic pressure volume relationships (Bull *et al*. 2016; Hinze *et al*. 2016) caused by increased passive stiffness of cardiomyocytes (Bull *et al*. 2016) and myofibrils (Elhamine *et al*. 2014). Stiffer titin isoforms lead to increased kinetics of contraction in myofibril experiments (Elhamine *et al*. 2014). In contrast to what is observed in mice with giant titin isoforms, these mice have a reduced exercise capacity (Slater *et al*. 2017).

Pre‐activation through increased end‐diastolic force (or passive force in skinned cardiomyocytes) has been proposed previously for skeletal muscle (Granzier & Wang, 1993) and cardiac muscle (Cazorla *et al*. 2001). Multiple studies have shown that the level of passive force determines the amount of length‐dependent activation (Granzier & Wang, 1993; Cazorla *et al*. 2001; Terui *et al*. 2008) and that titin plays an important role in this regulation (Terui *et al*. 2008). Consistent with previous data (Methawasin *et al*. 2014), developed force was less dependent on SL in HET than in WT cardiomyocytes (Fig. 3 *B*). However, the relationship between end‐diastolic force and developed force was not different between WT and HET cells (Fig. 3 *C*). This indicates that it is not SL *per se* that determines LDA, but rather the level of end‐diastolic force that is generated.

How the level of end‐diastolic force determines the developed force of the subsequent contraction (pre‐activation) is not exactly understood. From this and other studies it is clear that titin plays an important role. It has been suggested that stretching increases the ordering of myosin heads on the thin filament (Farman *et al*. 2011) or through titin‐based ordering of troponin C (Ait‐Mou *et al*. 2016). Another possible mechanism is through load‐dependent switching of myosin from the inactive OFF position to the force generating ON position that has recently been shown in cardiac muscle (Reconditi *et al*. 2017). Also, a more direct role for titin in active force development, through unfolding and refolding of its IgG domains, has been proposed (Rivas‐Pardo *et al*. 2016). The findings in the current paper are consistent with all these mechanisms.

Increases in titin compliance are not only caused by RBM20 mutations. They are a common feature in cardiac disease. Increased levels of the more compliant N2BA isoform (usually expressed as the N2BA:N2B ratio) are seen in most human disease samples. Patients with end‐stage heart failure due to ischaemic heart disease (Neagoe *et al*. 2002), dilated cardiomyopathy (Makarenko *et al*. 2004; Nagueh *et al*. 2004; Bollen *et al*. 2017a), heart failure with preserved ejection fraction (Borbely *et al*. 2009), peripartum cardiomyopathy (Bollen *et al*. 2017a) and hypertrophic cardiomyopathy (Nijenkamp, 2018) all show increased N2BA:N2B ratios. This increase in compliant titin isoform expression is usually considered to serve as compensation for the increased fibrosis seen in these disease states. Intriguingly, it could also be a way to cope with the high levels of diastolic calcium seen in end‐stage disease.

## Additional information

### Competing interests

None declared.

### Author contributions

AN, MH & DK designed the study. AN, MvdL, MS, PS, IB & MG collected data. AN, MS, PS & MvW analyzed the data. AN, PS, CO, JvdV, MH & DK interpreted the data. AN, MH & DK wrote the first draft of the manuscript. PS & JvdV critically revised the manuscript. All authors have read and approved the final version of this manuscript and agree to be accountable for all aspects of the work in ensuring that questions related to the accuracy or integrity of any part of the work are appropriately investigated and resolved. All persons designated as authors qualify for authorship, and all those who qualify for authorship are listed.
